# Age at menopause and all-cause and cause-specific dementia: a prospective analysis of the UK Biobank cohort

**DOI:** 10.1093/humrep/dead130

**Published:** 2023-06-21

**Authors:** Wenting Hao, Chunying Fu, Caiyun Dong, Chunmiao Zhou, Huizi Sun, Ziwei Xie, Dongshan Zhu

**Affiliations:** Centre for Health Management and Policy Research, School of Public Health, Cheeloo College of Medicine, Shandong University, Jinan, China; NHC Key Lab of Health Economics and Policy Research, Shandong University, Jinan, China; Department of Epidemiology, School of Public Health, Cheeloo College of Medicine, Shandong University, Jinan, China; Department of Epidemiology, School of Public Health, Cheeloo College of Medicine, Shandong University, Jinan, China; Department of Epidemiology, School of Public Health, Cheeloo College of Medicine, Shandong University, Jinan, China; Department of Epidemiology, School of Public Health, Cheeloo College of Medicine, Shandong University, Jinan, China; Department of Epidemiology, School of Public Health, Cheeloo College of Medicine, Shandong University, Jinan, China; Department of Epidemiology, School of Public Health, Cheeloo College of Medicine, Shandong University, Jinan, China

**Keywords:** age, natural menopause, surgical menopause, dementia, Alzheimer’s disease

## Abstract

**STUDY QUESTION:**

Are there associations between natural or surgical menopause and incident dementia by age at menopause?

**SUMMARY ANSWER:**

Compared to age at menopause of 46–50 years, earlier natural menopause (≤40 and 41–45 years) was related to higher risk of all-cause dementia, while a U-shape relationship was observed between age at surgical menopause and risk of dementia.

**WHAT IS KNOWN ALREADY:**

Menopause marks the end of female reproductive period. Age at menopause reflects the length of exposure to endogenous estrogen. Evidence on the association between age at natural, surgical menopause, and risk of dementia has been inconsistent.

**STUDY DESIGN, SIZE, DURATION:**

A population-based cohort study involving 160 080 women who participated in the UK Biobank study.

**PARTICIPANTS/MATERIALS, SETTING, METHODS:**

Women with no dementia at baseline, and had no missing data on key exposure variables and covariates were included. Cox proportional hazards models were used to estimate hazard ratios (HRs) and 95% CIs on the association of categorical menopause age with incident all-cause dementia, Alzheimer’s disease (AD) and vascular dementia (VD). Restricted cubic splines were used to model the non-linear relationship between continuous age at natural, surgical menopause, and risk of dementia. In addition, we analyzed the interaction effect of ever-used menopausal hormone therapy (MHT) at baseline, income level, leisure activities, and age at menopause on risk of dementia.

**MAIN RESULTS AND THE ROLE OF CHANCE:**

Compared to women with age at menopause of 46–50 years, women with earlier natural menopause younger than 40 years (1.36, 1.01–1.83) and 41–45 years (1.19, 1.03–1.39) had a higher risk of all-cause dementia, while late natural menopause >55 years was linked to lower risk of dementia (0.83, 0.71–0.98). Compared to natural menopause, surgical menopause was associated with 10% higher risk of dementia (1.10, 0.98–1.24). A U-shape relationship was observed between surgical menopause and risk of dementia. Women with surgical menopause before age 40 years (1.94, 1.38–2.73) and after age 55 years (1.65, 1.21–2.24) were both linked to increased risk of all-cause dementia. Women with early natural menopause without ever taking MHT at baseline had an increased risk of AD. Also, in each categorized age at the menopause level, higher income level or higher number of leisure activities was linked to a lowers risk of dementia.

**LIMITATIONS, REASONS FOR CAUTION:**

Menopausal age was based on women’s self-report, which might cause recall bias.

**WIDER IMPLICATION OF THE FINDINGS:**

Women who experienced natural menopause or had surgical menopause at an earlier age need close monitoring and engagement for preventive health measures to delay the development of dementia.

**STUDY FUNDING/COMPETING INTERESTS:**

This work was supported by the Start-up Foundation for Scientific Research in Shandong University (202099000066), Science Fund Program for Excellent Young Scholars of Shandong Provence (Overseas) (2022HWYQ-030), and the National Natural Science Foundation of China (82273702). There are no competing interests.

**TRIAL REGISTRATION NUMBER:**

N/A.

## Introduction

With aging and the increase in life expectancy, dementia has become a great global public health challenge (Winblad *et al.*, 2016). There were more than 50 million people living with dementia worldwide, and the number will reach 152 million by 2050 (Alzheimer’s Disease International, 2018). About two-thirds of Alzheimer’s disease (AD) patients are women (Prince *et al.*, 2016; Sindi *et al.*, 2021), and it is not just because women live longer and have more chances to develop AD. Common societal and lifestyle risk factors, such as education, exercise, smoking, and alcohol use, also cannot fully explain the sex difference (Podcasy and Epperson, 2016; Langa *et al.*, 2017; Mielke, 2018). The reasons why women are more likely to develop AD remain less clear. Some female-specific risk factors, such as menopause and menopausal hormone therapy (MHT), might be related to the elevated risk of dementia in women.

Menopause marks the end of female reproductive period. Age at menopause reflects the length of exposure to endogenous estrogen. Compared to men of the same age, postmenopausal women had 1.5–3.0 times higher risk of AD (Morrison *et al.*, 2006). The association between age at menopause and risk of dementia has been inconsistent. A recent systematic review that pooled findings from observational studies showed that later menopause was linked to a lower risk of all-cause dementia and AD (Fu *et al.*, 2022). Another review found no association between menopausal age (later versus younger) and risk of dementia (Georgakis *et al.*, 2016). Both of these two reviews did not separate natural and surgical menopause, and moderate heterogeneities among studies were observed when pooled estimates in these reviews. A specific review on surgical menopause and risk of dementia found surgical menopause at ≤45 years of age was associated with higher risk of dementia, compared to surgical menopause after age 45 years (Georgakis *et al.*, 2019). Results also have been contradicted from single large-scale or long-term follow-up cohort studies (Najar *et al.*, 2020; Yoo *et al.*, 2020). Thus, additional large-scale cohort studies are necessary to replicate these findings.

Menopause concurs with the marked decline of endogenous estrogen. During menopause, women may take menopause hormone therapy (MHT) to reduce their vasomotor symptoms (Stuenkel, 2015). The association of menopausal age with dementia might be moderated by the use of MHT (Stute *et al.*, 2021). In addition, Gong *et al.* (2022) found that lower SES level might strengthen the relationship of early menopause with dementia, and no clear interaction between smoking and menopause was observed. A study that involved 3568 people from Latin America did not observe an interaction between APOE genotype (none versus one or more APOE e4 alleles) and reproductive period (Prince *et al.*, 2018). Thus, whether socioeconomic status (education and income level), behavior (smoking status and leisure activities), and genetic factors (apolipoprotein E (APOE) allele status) modify the association between age at menopause and dementia is worth a further analysis. Based on the above mentioned, we first aimed to examine the association between natural, surgical menopause and risk of all-cause dementia, AD, and vascular dementia (VD) by age at menopause. Then, we examined whether there was an interaction effect between MHT used or not at baseline, socioeconomic status, behavior, genetic factors, and age at menopause on risk of dementia.

## Materials and methods

### Study design and participants

**Figure 1.**
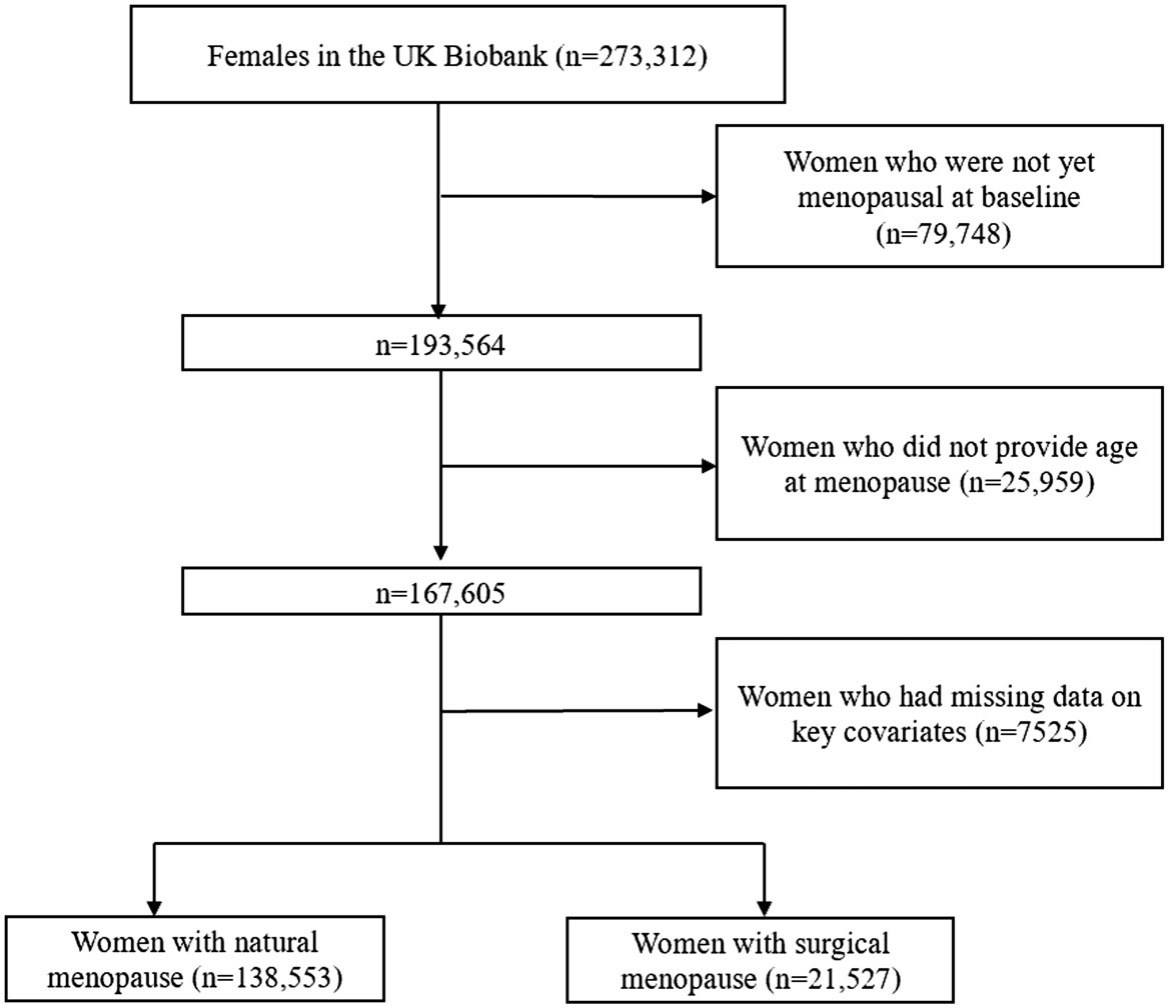
Flow diagram of study participant selection.

The study subjects came from the UK Biobank. The UK Biobank is a large population-based cohort study established in the UK from 2006 to 2010 (Sudlow *et al.*, 2015). At recruitment, participants with an age range from 40 to 71 years old provided electronically signed consent forms, answered questions about sociodemographic, lifestyle, and health-related factors, and completed a series of physical measurements. All participants were linked to hospital data and national death registries from England, Scotland, and Wales (Gong *et al.*, 2021) to determine the date of the first diagnosis of dementia after the baseline assessment. Totally, 1297 people were loss to follow-up because they had left the UK or had withdrawn consent for future linkage. UK Biobank received ethical approval from the UK National Health Service’s National Research Ethics Service (ref 11/NW/0382). This research was conducted under UK Biobank application number 68369. A prospective analysis was adopted based on postmenopausal women with no dementia at baseline, who had no missing data on key covariates, including ethnicity, education, income, cigarette smoking, alcohol drinking, leisure activities, BMI, cardiovascular disease (CVD) history, the E4 variant of apolipoprotein E (APOE e4), and MHT used status at baseline. A total of 160 080 women were included (Fig. 1).

### Exposure variables and outcome variables

In the present study, natural menopause was defined as an absence of menstruation over a period of 12 months and no experience of hysterectomy and/or oophorectomy before it. Surgical menopause was defined as the removal of both ovaries (bilateral oophorectomy) before natural menopause. Age at menopause was categorized as ≤40 (premature), 41–45 (early), 46–50, 51–55, and >55 years (late menopause). The primary outcome variable in this study was the first occurrence of all-cause dementia, including AD and VD. Physician-diagnosed dementia was ascertained from linkage data to primary care, hospital admission, and death register records. The International Classification of Diseases 10th Edition (ICD-10) codes F00, F01, F02, F03, G30, G31.0, G31.8, and ICD-9 code 290 were used to identify participants with all-cause dementia if one or more of these codes were recorded as a primary or secondary diagnosis. Incident AD was defined by ICD-10 codes F00, G30, and ICD-9 code 290. Incident VD was defined by ICD-10 code F01. Outcome adjudication for incident dementia was conducted by the UK Biobank Outcome Adjudication team.

### Covariates

We included the following factors in the analyses as covariates because these have been shown to be associated with both age at menopause (Schoenaker *et al.*, 2014; Zhu *et al.*, 2019) and risk of dementia (Dong *et al.*, 2022; Menesgere *et al.*, 2023): age at baseline, ethnicity, education level, income, BMI, smoking status, physical activities, drinking status, leisure activities, CVD, APOE e4 carrier status, and ever-used MHT at baseline. Ethnicity was divided into white and non-white. Years of education were categorized as ≤10, 11–12, and >12 years. Total household income before tax was split into three groups: <£18 000, £18 000–30 999, and ≥£31 000. BMI was classified as <18.5, 18.5–24.9, 25–29.9, and ≥30 kg/m^2^. Cigarette smoking was classified as smoking almost all days, occasionally, ex-smoker, and never smoked. International Physical Activity Questionnaire (IPAQ) was used to calculate metabolic equivalent (MET) score, and physical activity level was categorized as low (<600 MET-min/week), medium (600–3000 MET-min/week), and high (≥3000 MET-min/week). Alcohol intake was categorized as daily, 3–4 times/week, 1–2 times/week, occasionally, and never. Leisure activities were assessed through participants’ choices of six activities and were categorized as none, one, and two or more. CVD history was classified into yes or no. APOE allele status was based on two single nucleotide polymorphisms: rs7412 and rs429358. Participants with APOE e4 allele (e3/e4, e4/e4, and occasionally e2/e4 genotypes) were compared with those with the e2/e2, e2/e3, or e3/e3 genotype. MHT used status was collected at baseline through a question ‘Have you ever used hormone replacement therapy (HRT)?’ and was classified as user or non-user. Detailed information on covariates collection and definitions is listed in Supplementary Table S1.

### Statistical analysis

Baseline characteristics were presented as means and SD for continuous variables and as percentages (%) for categorical variables. Cox proportional hazard risk model was used to estimate hazard ratios (HR) and 95% CIs between age at menopause and risk of all-cause dementia, AD, and VD. When modeling the association with AD or VD, only those with AD or VD were included, i.e. women who had both AD and VD were excluded. Data were censored on 30 January 2021 or at the date of death. For women who experienced dementia, follow-up time was calculated as their age at diagnosis of dementia minus baseline age. For participants without experiencing dementia, follow-up time was defined as their age at the last follow-up (censored date) minus baseline age. When types of menopause were compared, women with natural menopause were used as the reference group, and when age at menopause was analyzed, women with age at menopause 46–50 years were used as the reference group. Covariates were adjusted sequentially, i.e. in Model 1, sociodemographic factors were adjusted (age at baseline, ethnicity, years of education, and income level). As dementia incidence increases exponentially with age and doubles about every 5 years beyond age 65, when age at baseline was adjusted, we used a quadratic form of age. In Model 2, lifestyle factors were further adjusted (smoking status, physical activity, alcohol consumption, leisure activities, and BMI level). In Model 3, CVD history and APOE e4 allele status were further adjusted. In Model 4, ever-used MHT at baseline was further adjusted. As CVD might be a mediator between age at menopause and dementia, we did analyses to see the changes in HR (95% CI) before and after CVD was adjusted. Also, we performed two sensitivity analyses, i.e. first only including dementia which occurred at least 5 years after menopause, and second by excluding women with ever-used MHT at baseline. Furthermore, we used a restricted cubic spline to model the non-linear relationships between continuous age at natural menopause, age at surgical menopause, and risk of dementia, with covariates being adjusted as in Model 4. In addition, we examined the interaction effect of MHT used status at baseline, education, income, smoking, leisure activities, and APOE e4 carrier status with categorized age at menopause by adding a product interaction term to Model 4. A two-sided *P* value of 0.05 or less indicated the significance of the interaction effect.

We used SAS (version 9.4, SAS Institute Inc, Cary, NC) in all statistical analyses. The PHREG procedure was used to fit the Cox proportional hazards regression models. All statistical tests were based on the two-sided 5% level of significance.

## Results

### Characteristics of the participants

There were 160 080 women in this study, 138 553 with natural menopause and 21 527 with surgical menopause. The mean (SD) age at baseline was 59.2 ± 6.1 years, with a range from 40 to 71 years old. The mean (SD) age at the last follow-up was 72.1 ± 6.1 years. After a mean (SD) follow-up time of 12.1 ± 1.6 years from baseline, there were 1.8% (2427), 11.8% (16 279), and 9.9% (13 722) women with premature, early, and late natural menopause, respectively. The cumulative incidences of all-cause dementia, AD, and VD were 1.32% (1824, 27.3% cases from death certificates), 0.61% (841), and 0.25% (353), respectively. Compared with women without dementia, women with dementia were more likely to be less educated, obese and to be current smokers, with CVD (Table 1). Characteristics of women with surgical menopause were listed in Supplementary Table S2. Compared to women who were included, those excluded for missing age at menopause were relative older at baseline (mean age 61.7 versus 59.2 years) and had higher proportion of MHT used at baseline (65.3% versus 41.6%) (Supplementary Table S3). Women who were excluded for missing covariates had higher cumulative incidence of dementia (2.2% versus 1.3%) (Supplementary Table S4).

**Table 1.** Characteristics by age at natural menopause and incident all-cause dementia events.

	Age at natural menopause					Incident dementia	
Characteristics	≤40 years	41–45 years	46–50 years	51–55 years	≥55 years	No	Yes
	(n = 2427)	(n = 16 279)	(n = 47 092)	(n = 59 033)	(n = 13 722)	(n = 136 729)	(n = 1824)
Age at baseline	57.0 ± 7.7	58.7 ± 7.0	58.6 ± 6.3	59.3 ± 5.6	61.6 ± 4.6	59.1 ± 6.0	64.5 ± 3.4
Ethnicity							
White	2280 (93.9)	15 342 (94.2)	44 869 (95.3)	57 083 (96.7)	13 331 (97.2)	131 139 (95.9)	1766 (96.8)
Nonwhite	147 (6.1)	937 (5.8)	2223 (4.7)	1950 (3.3)	391 (2.8)	5590 (4.1)	58 (3.2)
Education level							
≤10	1420 (58.5)	8983 (55.3)	24 090 (51.2)	27 699 (46.9)	6938 (50.6)	67 989 (49.7)	1141 (62.6)
11–12	270 (11.1)	1797 (11.0)	5532 (11.7)	7473 (12.7)	1532 (11.1)	16 422 (12.0)	182 (10.0)
>12	737 (30.4)	5499 (33.7)	17 470 (37.1)	23 861 (40.4)	5252 (38.3)	52 318 (38.3)	501 (27.4)
Income (£)							
<18 000	776 (32.0)	4974 (30.6)	12 690 (27.0)	14 563 (24.7)	3940 (28.7)	40 326 (27.5)	516 (46.1)
18 000–30 999	592 (24.4)	4322 (26.6)	12 612 (26.8)	15 975 (27.1)	3858 (28.1)	40 266 (27.4)	304 (27.1)
≥31 000	1059 (43.6)	6983 (42.8)	21 790 (46.2)	28 495 (48.2)	5924 (43.2)	66 196 (45.1)	300 (26.8)
BMI							
<18.5 kg/m^2^	37 (1.5)	158 (1.0)	414 (0.9)	406 (0.7)	77 (0.6)	1076 (0.8)	16 (0.9)
18.5–24.9 kg/m^2^	874 (36.0)	6239 (38.3)	19 282 (41.0)	23 793 (40.3)	4767 (34.7)	54 293 (39.7)	662 (36.3)
25.0–29.9 kg/m^2^	848 (35.0)	6070 (37.3)	17 447 (37.1)	22 316 (37.8)	5379 (39.2)	51 392 (37.6)	668 (36.6)
≥30kg/m^2^	668 (27.5)	3812 (23.4)	9949 (21.0)	12 518 (21.2)	3499 (25.5)	29 968 (21.9)	478 (26.2)
Cigarette smoking							
Never smoker	1225 (50.5)	8602 (52.8)	27 047 (57.4)	36 118 (61.2)	8280 (60.3)	80 293 (58.7)	603 (53.8)
Former smoker	820 (33.8)	5685 (34.9)	15 719 (33.4)	19 219 (32.6)	4727 (34.5)	45 493 (33.3)	423 (37.8)
Current smoker	382 (15.7)	1992 (12.3)	4326 (9.2)	3696 (6.2)	715 (5.2)	10 943 (8.0)	94 (8.4)
Alcohol drinking							
Never drinker	198 (8.2)	1136 (7.0)	2682 (5.7)	2856 (4.8)	687 (5.0)	8166 (5.6)	125 (11.2)
Former drinker	132 (5.4)	653 (4.0)	1810 (3.8)	1795 (3.0)	450 (3.3)	5233 (3.6)	74 (6.6)
Current drinker	2097 (86.4)	14 490 (89.0)	42 600 (90.5)	54 382 (92.2)	12 585 (91.7)	133 389 (90.8)	921 (82.2)
No. of leisure activities							
0	806 (33.2)	4795 (29.5)	12 971 (27.5)	14 510 (24.6)	3249 (23.6)	35 757 (26.2)	574 (31.5)
1	985 (40.6)	7027 (43.1)	19 823 (42.1)	24 402 (41.3)	5660 (41.3)	57 092 (41.8)	805 (44.1)
≥2	636 (26.2)	4457 (27.4)	14 298 (30.4)	20 121 (34.1)	4813 (35.1)	43 880 (32.0)	445 (24.4)
CVD							
No	2221 (91.5)	15 130 (92.9)	44 595 (94.7)	56 240 (95.3)	12 900 (94.0)	129 604 (94.8)	1482 (81.3)
Yes	206 (8.5)	1149 (7.1)	2497 (5.3)	2793 (4.7)	822 (6.0)	7125 (5.2)	342 (18.7)
APOE e4							
No APOE e4	1807 (74.5)	12 374 (76.0)	35 849 (76.1)	44 744 (75.8)	10 353 (75.5)	104 188 (76.2)	939 (51.5)
One APOE e4	565 (23.3)	3588 (22.0)	10 303 (21.9)	13 113 (22.2)	3092 (22.5)	29 956 (21.9)	705 (38.7)
Two APOE e4	55 (2.2)	317 (2.0)	940 (2.00)	1176 (2.0)	277 (2.0)	2585 (1.9)	180 (9.8)
Ever-used MHT at baseline							
No	802 (33.0)	7899 (48.5)	28 276 (60.0)	36 454 (61.8)	7413 (54.0)	79 952 (58.5)	892 (48.9)
Yes	1625 (67.0)	8380 (51.5)	18 816 (40.0)	22 579 (38.2)	6309 (46.0)	56 777 (41.5)	932 (51.1)

CVD, cardiovascular disease; APOE, apolipoprotein E; MHT, menopausal hormone therapy.

### Age at natural menopause and dementia

**Figure 2.**
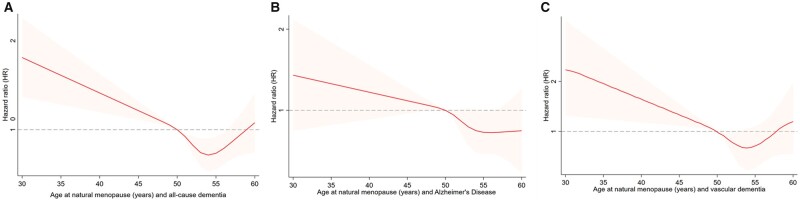
**Non-linear relationships between age at natural menopause and dementia.** (**A**) all-cause dementia, (**B**) Alzheimer’s disease, and (**C**) vascular dementia.

Compared to women with age at natural menopause 46–50 years, women with premature menopause (≤40 years) and early menopause (41–45 years) had a higher risk of all-cause dementia, with HR (95% CI) of 1.36 (1.01, 1.83), and 1.19 (1.03, 1.37), respectively, while women with menopausal age 51–55 years and late menopause (>55 years) were linked to lower risk of dementia, with HR (95% CI) of 0.83 (0.74, 0.92) and 0.83 (0.71, 0.98), respectively. When the associations with AD and VD were analyzed separately, we found the HRs in earlier and later menopause groups had the same direction as the HRs with all-cause dementia, although the 95% CIs spanned one (Table 2; Supplementary Table S5). When CVD was adjusted, the HR values were slightly weakened (Supplementary Table S6). Sensitivity analyses that only included dementia which occurred 5 years after menopause or only included women without ever-used MHT at baseline showed similar findings to Table 2 (Supplementary Tables S7 and S8). When restricted cubic splines were used to model the non-linear relationships between continuous age at natural menopause and dementia, we found that the dose–response relationship was consistent with the trend of association when categorical age of menopause was used (Fig. 2).

**Table 2.** Associations between age at natural menopause and all-cause dementia, Alzheimer's disease (AD) and vascular dementia (VD).

Dementia	Years	Women	Dementia	Incidence rate (per 10 000 person-years)	Model 1	Model 2	Model 3	Model 4
					HR (95% CI)	HR (95% CI)	HR (95% CI)	HR (95% CI)
All-cause dementia	≤40	2427	49	16.9	1.60 (1.20, 2.15)	1.51 (1.13, 2.03)	1.37 (1.02, 1.84)	1.36 (1.01, 1.83)
	41–45	16 279	289	14.6	1.25 (1.09, 1.44)	1.23 (1.07, 1.42)	1.20 (1.04, 1.38)	1.19 (1.03, 1.37)
	46–50	47 092	634	11.1	1	1	1	1
	51–55	59 033	653	9.1	0.80 (0.71, 0.89)	0.82 (0.73, 0.91)	0.83 (0.74, 0.92)	0.83 (0.74, 0.92)
	>55	13 722	199	12.0	0.81 (0.69, 0.95)	0.83 (0.71, 0.98)	0.84 (0.71, 0.98)	0.83 (0.71, 0.98)
Alzheimer’s disease (AD)	≤40	2427	24	8.3	1.71 (1.13, 2.59)	1.63 (1.07, 2.47)	1.49 (0.98, 2.27)	1.48 (0.98, 2.25)
	41–45	16 279	118	6.0	1.08 (0.87, 1.34)	1.06 (0.86, 1.32)	1.04 (0.84, 1.29)	1.04 (0.84, 1.29)
	46–50	47 092	300	5.3	1	1	1	1
	51–55	59 033	314	4.4	0.81 (0.69, 0.95)	0.83 (0.71, 0.97)	0.84 (0.71, 0.98)	0.84 (0.72, 0.98)
	>55	13 722	85	5.1	0.73 (0.57, 0.93)	0.74 (0.58, 0.95)	0.75 (0.58, 0.95)	0.74 (0.58, 0.95)
Vascular dementia (VD)	≤40	2427	12	4.1	1.99 (1.10, 3.60)	1.84 (1.01, 3.33)	1.61 (0.89, 2.92)	1.59 (0.88, 2.88)
	41–45	16 279	62	3.2	1.35 (0.99, 1.83)	1.31 (0.96, 1.79)	1.26 (0.93, 1.72)	1.25 (0.92, 1.70)
	46–50	47 092	122	2.1	1	1	1	1
	51–55	59 033	120	1.7	0.76 (0.59, 0.98)	0.79 (0.61, 1.02)	0.80 (0.62, 1.03)	0.80 (0.62, 1.03)
	>55	13 722	37	2.2	0.75 (0.52, 1.08)	0.77 (0.53, 1.12)	0.78 (0.54, 1.13)	0.78 (0.54, 1.12)

Model 1: age at baseline, ethnicity, BMI, education level, and income level were adjusted; Model 2: leisure activities, cigarette smoking, and alcohol drinking were further adjusted based on Model 1; Model 3: cardiovascular disease (CVD) and APOE (apolipoprotein E) were further adjusted based on Model 2; Model 4: ever-used menopausal hormone therapy (MHT) at baseline was further adjusted based on Model 3. HR, hazard ratio.

### Surgical menopause and dementia

**Figure 3.**
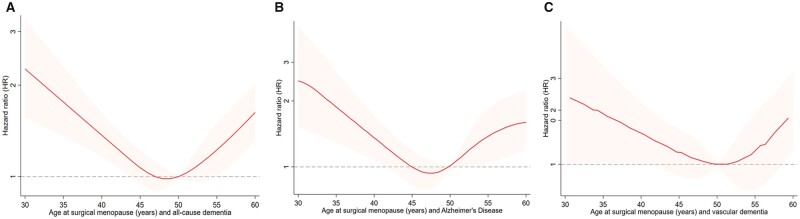
**Non-linear relationships between age at surgical menopause and dementia.** (**A**) all-cause dementia, (**B**) Alzheimer’s disease, and (**C**) vascular dementia.

Compared to natural menopause, surgical menopause was associated with 10% higher risk of dementia (HR 1.10, 95% CI 0.98–1.24) (Table 3). Women with surgical menopause before age 40 years and after age 55 years were linked to an increased risk of all-cause dementia, with HR (95% CI) of 1.94 (1.38, 2.73) and 1.65 (1.21, 2.24), respectively. When the associations with AD and VD were analyzed, surgical menopause before age 40 years was linked to higher risk of AD (2.16, 1.32–3.54) and surgical menopause after age 55 was linked to higher risk of VD (1.95, 1.04–4.02) (Table 4). Restricted cubic spline models showed a U-shape relationship between continuous surgical menopause and risk of dementia (Fig. 3).

**Table 3.** Associations between menopause type (natural menopause versus surgical menopause) and all-cause dementia, Alzheimer's disease (AD), and vascular dementia (VD).

Dementia	Type	Women	Dementia	Model 1	Model 2	Model 3	Model 4
All cause dementia	Natural	138 553	1824	1	1	1	1
	Surgical	21 527	360	1.17 (1.04, 1.31)	1.15 (1.03, 1.29)	1.11 (0.99, 1.25)	1.10 (0.98, 1.24)
Alzheimer's disease (AD)	Natural	138 553	841	1	1	1	1
	Surgical	21 527	163	1.16 (0.98, 1.38)	1.15 (0.97, 1.36)	1.11 (0.93, 1.31)	1.11 (0.93, 1.33)
Vascular dementia (VD)	Natural	138 553	353	1	1	1	1
	Surgical	21 527	64	1.03 (0.78, 1.35)	1.01 (0.77, 1.33)	0.96 (0.73, 1.26)	0.93 (0.71, 1.23)

Model 1: age at baseline, ethnicity, BMI, education level, income level and age at menopause were adjusted; Model 2: leisure activities, cigarette smoking, and alcohol drinking were further adjusted based on Model 1; Model 3: cardiovascular disease (CVD) and APOE (apolipoprotein E) were further adjusted based on Model 2; Model 4: ever-used menopausal hormone therapy (MHT) at baseline was further adjusted based on Model 3. HR, hazard ratio.

**Table 4.** Associations between age at surgical menopause and all-cause dementia, Alzheimer's disease (AD), and vascular dementia (VD).

Dementia	Years	Women	Dementia	Incidence rate (per 10 000 person-years)	Model 1	Model 2	Model 3	Model 4
					HR (95% CI)	HR (95% CI)	HR (95% CI)	HR (95% CI)
All-cause dementia	≤40	2862	64	18.5	2.23 (1.59, 3.13)	2.08 (1.48, 2.92)	1.98 (1.41, 2.79)	1.94 (1.38, 2.73)
	41–45	4945	53	8.9	1.06 (0.74, 1.50)	1.02 (0.72, 1.46)	1.00 (0.70, 1.42)	1.00 (0.70, 1.42)
	46–50	6238	79	10.5	1	1	1	1
	51–55	3754	58	12.9	1.19 (0.85, 1.68)	1.20 (0.85, 1.70)	1.20 (0.85, 1.68)	1.16 (0.82, 1.63)
	>55	3728	106	24.1	1.74 (1.29, 2.35)	1.74 (1.29, 2.34)	1.80 (1.33, 2.42)	1.65 (1.21, 2.24)
Alzheimer's disease (AD)	≤40	2862	30	8.7	2.34 (1.44, 3.82)	2.25 (1.38, 3.67)	2.24 (1.37, 3.66)	2.16 (1.32, 3.54)
	41–45	4945	19	3.2	0.81 (0.47, 1.42)	0.80 (0.46, 1.39)	0.78 (0.45, 1.37)	0.78 (0.45, 1.37)
	46–50	6238	37	4.9	1	1	1	1
	51–55	3754	34	7.6	1.44 (0.90, 2.30)	1.46 (0.91, 2.33)	1.44 (0.90, 2.31)	1.38 (0.86, 2.21)
	>55	3728	43	9.8	1.45 (0.93, 2.27)	1.46 (0.93, 2.28)	1.54 (0.99, 2.42)	1.37 (0.87, 2.17)
Vascular dementia (VD)	≤40	2862	11	3.2	2.42 (1.06, 5.50)	2.24 (0.98, 5.14)	1.84 (0.80, 4.22)	1.79 (0.78, 4.11)
	41–45	4945	8	1.3	1.02 (0.42, 2.49)	0.97 (0.40, 2.39)	0.91 (0.37, 2.23)	0.91 (0.37, 2.22)
	46–50	6238	12	1.6	1	1	1	1
	51–55	3754	11	2.4	1.43 (0.63, 3.25)	1.47 (0.65, 3.33)	1.45 (0.64, 3.28)	1.40 (0.62, 3.20)
	>55	3728	22	5.0	2.12 (1.04, 4.31)	2.11 (1.04, 4.31)	2.11 (1.03, 4.31)	1.95 (1.04, 4.02)

Model 1: age at baseline, ethnicity, BMI, education level, and income level were adjusted; Model 2: leisure activities, cigarette smoking, and alcohol drinking were further adjusted based on Model 1; Model 3: cardiovascular disease (CVD) and APOE (apolipoprotein E) were further adjusted based on Model 2; Model 4: ever-used menopausal hormone therapy (MHT) at baseline was further adjusted based on Model 3. HR, hazard ratio.

### The roles of ever-used MHT at baseline, income, and leisure activities

After a formal test, there was a significant interaction between age at natural menopause and MHT used or not at baseline, income, and leisure activities. Women with early natural menopause without taking MHT at baseline had an increased risk of AD (1.36, 1.03–1.81), while no such relationship was observed in those taking MHT at baseline (1.03, 0.78–1.37) (Supplementary Fig. S1). In each age at the menopause categorized level, higher income level or higher number of leisure activities was linked to lower risk of dementia (Supplementary Figs S2 and S3).

## Discussion

Our findings showed that compared to age at menopause of 46–50 years, earlier natural menopause (≤40 and 41–45 years) was related to higher risk of all-cause dementia, while later menopause (51–55 and >55 years) was linked to lower risk of dementia. There was a U-shape relationship between age at surgical menopause and risk of all-cause dementia. Income level, number of leisure activities, and whether used MHT at baseline modified the relationship between menopausal age and risk of dementia.

### Age at natural menopause and all-cause dementia

Evidence on the association between age at natural menopause and all-cause dementia has been inconsistent (Geerlings *et al.*, 2001; Prince *et al.*, 2018; Yoo *et al.*, 2020). A recent systematic review that pooled findings from 10 observational studies (involving over 4.7 million) showed that early menopause (<45 years) was linked to higher risk of all-cause dementia and AD, and dose–response meta-analyses also showed a linear relationship that the younger menopause, the higher risk of dementia. Nevertheless, this review did not distinguish natural or surgical menopause, and moderate heterogeneity was observed among studies (Fu *et al.*, 2022). Another review included 13 observational studies (19 449 women were involved) that found no association between menopausal age (later versus younger) and risk of dementia, also with great heterogeneity among studies (Georgakis *et al.*, 2016). However, when pooling the estimates, this review did not use uniform classification for age at menopause, and the reference level differed across studies (Georgakis *et al.*, 2016). Recent large-scale or long-term follow-up cohort studies also showed contradicted findings. In a nationwide cohort study which involved 4.7 million Korean postmenopausal women, Yoo *et al.* reported that women with late menopause (≥55 years) had 21% lower risk of dementia compared to those who had menopause at age <40 years. However, this study used data from national health insurance service, and some key covariates, e.g. ever-used MHT at baseline, APOE e4 carrier status, and education level, were not available, which might cause some bias (Yoo *et al.*, 2020). In contrast, a 44-year longitudinal study of Swedish postmenopausal women found that each 1 year later of menopause age was associated with 7% higher risk of dementia (1.07, 1.04–1.10). However, this study included postmenopausal women before the year 2002. The proportion of MHT prescriptions prior to 2002 was much higher than its prescription after 2002 (Zbuk and Anand, 2012), which may affect the association between menopausal age and dementia in a real-world context. Consistent with previous studies (Gilsanz *et al.*, 2019; Yoo *et al.*, 2020) after a series of covariates were adjusted, we observed that later menopause was linked to lower risk of all-cause dementia.

### Age at natural menopause and AD

The association between age at menopause and risk of AD is also unclear (Yamada *et al.*, 2009; Najar *et al.*, 2020; Yoo *et al.*, 2020). In Korean women, Yoo *et al.* (2020) found that later menopause (≥55 versus <40 years) was related to 20% lower risk of AD, while Najar *et al.* (2020) have found an opposite finding, i.e. each year later of menopause was associated with 7% higher risk of AD. In addition, Yamada *et al.* (2009) did not observe an association between them. In UK females, compared to women who had menopause at 46–50 years, we observed late age at natural menopause (>55 years) was associated with lower risk of AD, consistent with a Korean study (Yoo *et al.*, 2020).

### Age at natural menopause and VD

Few studies have examined the association between age at menopause and VD (Yamada *et al.*, 2009; Yoo *et al.*, 2020). The adult health study in Japan found no association between menopause age and VD (Yamada *et al.*, 2009). The study in Korea found that the risk of VD decreased with the rise of age at menopause (Yoo *et al.*, 2020). VD is usually caused by diseases that disrupt the blood supply to the brain (Vijayan and Reddy, 2016), and stroke was acknowledged a major risk factor for VD (Desmond *et al.*, 2000; Ivan *et al.*, 2004; Gamaldo *et al.*, 2006). There has been a link between menopausal age and stoke. Compared to women with normal menopause, early menopause (<45 years) was linked to 50% higher risk of having stroke (Zhu *et al.*, 2019). In our findings, a trend that early menopause might be related to higher risk of VD was also observed.

### Surgical menopause and dementia

Evidence on the associations between surgical menopause and dementia risk remains limited. A review that included four studies and 12 731 people reported that overall surgical menopause was not associated with the risk of dementia (HR: 1.16, 95% CI: 0.96–1.43), but early surgical menopause (≤45 years) was associated with higher risk of dementia (1.70, 95% CI: 1.07–2.69) (Georgakis *et al.*, 2019). Nevertheless, this review did not consider the association of later surgical menopause with dementia. In a recent study using Danish Nurse Cohort which involved 24 851 women over 60 years old, Cecilie *et al.* found the association between surgery menopause and dementia was 1.18 (0.89–1.56), while this study did not categorize age at surgical menopause, and their findings were limited by the statistical power (Uldbjerg *et al.*, 2022). Using a broad categorization of age at surgical menopause, we found that compared to natural menopause, overall surgical menopause was linked to 10% higher risk of dementia. Besides, there was a U-shape relationship between age at surgical menopause and all-cause dementia risk. Both surgical menopause before 40 years and after 55 years were related to an elevated risk of dementia. We observed that women with later surgical menopause had an increased risk of dementia. However, this relationship should be interpreted with caution. This might be because people with later age at surgical menopause (e.g. ≥55 years) had elder age at baseline than people with later age at natural menopause (63.4 versus 61.6). Also, misclassifications of types of menopause may have occurred. Women who had earlier natural menopause before surgical menopause might not report their age at natural menopause and were classified into the surgical menopause group. Thus, the general trend for late surgical menopause and dementia might be artefactual.

### Mechanisms

Several mechanisms have been proposed to explain the association between early menopause and increased risk of dementia. The ‘estrogen hypothesis’ suggests that estrogen has a protective effect against AD dementia. During menopause, the decline in circulating estrogen coincides with a decrease in brain bioenergetics and a shift to a metabolically impaired phenotype in these brain regions (Rettberg *et al.*, 2014). Inadequate or absent compensatory bioenergetic adaptation to inadequate estrogen activation triggers not only hallmark symptoms of menopause but also cognitive changes that increase the risk of late-onset AD in postmenopausal women. Second, estrogen can activate cellular antioxidants, e.g. glutathione, to reduce Aβ deposition. Lack of estrogen in a long term enhances oxidative stress, which may increase neuronal aging and contribute to cognitive impairment. Studies have found that postmenopausal women have higher levels of Aβ than perimenopausal and premenopausal women (Rahman *et al.*, 2020). Besides, some common factors such as adverse CVD risk factors, which were associated with both early reproductive aging and dementia risk might drive the association between them (Zhu *et al.*, 2019).

### Ever-used MHT at baseline, socioeconomic status, behavior, and dementia

Evidence on the association of MHT with dementia remains mixed in previous studies. One systematic review and meta-analysis showed that post-MHT was not associated with the risk of all-cause dementia and AD (O'Brien *et al.*, 2014), while another review reported that MHT was linked to 8% higher risk of AD and 16% increased risk of all-cause dementia (Wu *et al.*, 2020). Previous studies did not consider age at menopause when analyzing the effect of MHT. After combining age at menopause and ever-used MHT at baseline, we did not find that taking MHT at baseline increased risk of dementia. A recent population-based cohort study also reported that MHT use in postmenopausal women was not associated with an increased risk of developing dementia (Vinogradova *et al.*, 2021). Income is an indicator of socioeconomic status, contributing in shaping cognitive reserve. Our findings indicated higher income level or higher number of leisure activities were linked to lowers risk of dementia across different ages at menopause. Low income and greater financial strain predict incident dementia (Röhr *et al.*, 2022). Socioeconomic conditions are also associated with cognitive development in early life as well as modifiable risk factors in adulthood that may trigger neuropathological processes (e.g. smoking, physical activity, alcohol consumption, obesity, hypertension, diabetes, loneliness) (Cha *et al.*, 2021). Leisure activities have been associated with lower risk of all-cause dementia, AD, and VD (Su *et al.*, 2022). We observed leisure activities had a synergistic effect with age at menopause on the risk of dementia. Participation in leisure activities means more social connection, social support, and receiving diverse cognitive stimuli, which are all conducive in increasing cognitive reserve.

### Strengths and limitations

The strengths of this study include the large sample size which enabled us to examine the associations with dementia subtypes and to perform subgroup or combined analyses. Second, dementia outcomes were ascertained from primary care, hospital admissions, and mortality data, avoiding bias from self-reported data, and have been validated in a previous study. One study demonstrating positive predictive value for all-cause dementia was 80–87% (Wilkinson *et al.*, 2019). Another study reported that the sensitivity and specificity of hospital dementia diagnoses were 78.0% and 92.0%, respectively (Sommerlad *et al.*, 2018). In addition, the interaction effects of menopausal age with MHT used or not at baseline, socioeconomic, and behavioral factors on dementia were also considered.

Our study also has several limitations. First, menopausal age was based on women’s self-report, which might cause recall bias. However, previous studies have proved that the validity and reproducibility of self-reported age at menopause were good (den Tonkelaar, 1997). Second, we used lifestyle factors (e.g. smoking status and alcohol intake), BMI, and MHT reported at baseline (mid-age) as covariates rather than treating them as time-varying covariates, which may cause some bias. Third, detailed definitions of some covariates were lacking (e.g. number of cigarettes smoked per day and alcohol types), and leisure activities included multiple activities without being separated for analysis. Fourth, women who took MHT at baseline before the occurrence of the last menstrual period might cause menses to continue and would artificially raise the reported age at menopause. This could be a source of misclassification in MHT users. Fifth, we lacked information on age initiation, type, and duration of MHT use. This may affect the interpretation of our results to some extent. Sixth, participants of the UK Biobank are relatively healthier (e.g. lower rates of smoking), well educated, and less deprived than the general UK population. This may limit the generalizability and extrapolation of our findings to the broader population. Last, the present study included mainly white women living in the UK who reached menopause while remaining dementia free at baseline; this may limit the generalizability of the findings to other races.

In summary, compared to age at the menopause of 46–50 years, premature (≤40 years) and early (41–45 years) natural menopause were related to increased risk of all-cause dementia. There was a U-shape relationship between age at surgical menopause and risk of dementia, i.e. both surgical menopause before age 40 and after age 55 years were linked to higher risk of dementia. In clinical practice, late-life women who experienced natural menopause or had surgical menopause at an earlier age need close monitoring and engagement for preventive health measures and early diagnosis of dementia.

## Supplementary Material

dead130_Supplementary_Figure_S1

dead130_Supplementary_Figure_S2

dead130_Supplementary_Figure_S3

dead130_Supplementary_Table_S1

dead130_Supplementary_Table_S2

dead130_Supplementary_Table_S3

dead130_Supplementary_Table_S4

dead130_Supplementary_Table_S5

dead130_Supplementary_Table_S6

dead130_Supplementary_Table_S7

dead130_Supplementary_Table_S8

## Data Availability

The data described in the manuscript will be made available for researchers who apply to use the UK Biobank data set by registering and applying at https://www.ukbiobank.ac.uk/enable-your-research/register.
