# Supplementary material for: Age at menopause and all-cause and cause-specific dementia: a prospective analysis of the UK Biobank cohort
Source: Hum Reprod. 2023 Jun 21;38(9):1746–54. doi: 10.1093/humrep/dead130 (PMC10663050; doi:10.1093/humrep/dead130)
Supplement: dead130_Supplementary_Figure_S1 [file dead130_supplementary_figure_s1.pdf]

Human Reproduction, 2023, 1–1
https://doi.org/10.1093/humrep/dead130
Supplementary Data

MHT	No. of events	Total	HR (95% CI)	P Value
**All cause dementia**	.			
<=45 years +MHT	178	10,075	1.24 (1.03, 1.49)	0.023
<=45 years +no MHT	160	8773	1.41 (1.16, 1.71)	0.0005
46-50 years + MHT	317	18,816	1.13 (0.96, 1.32)	0.1344
46-50 years + no MHT	317	28,276	reference	
51-55 years +MHT	326	22,579	0.93 (0.80, 1.09)	0.3924
51-55 years +no MHT	327	36,454	0.77 (0.66, 0.91)	0.0013
>55 years + MHT	111	6309	1.03 (0.83, 1.28)	0.7981
>55 years + no MHT	88	7413	0.77 (0.61, 0.98)	0.0342
**AD**	.			
<=45 years +MHT	70	10,075	1.03 (0.78, 1.37)	0.8299
<=45 years +no MHT	72	8773	1.36 (1.03, 1.81)	0.0322
46-50 years + MHT	149	18,816	1.11 (0.88, 1.40)	0.3734
46-50 years + no MHT	151	28,276	reference	
51-55 years +MHT	156	22,579	0.93 (0.74, 1.17)	0.5344
51-55 years +no MHT	158	36,454	0.78 (0.62, 0.98)	0.0314
>55 years+ MHT	47	6309	0.90 (0.65, 1.25)	0.5206
>55 years+ no MHT	38	7413	0.69 (0.48, 0.99)	0.0444
**VD**	.			
<=45 years +MHT	45	10,075	1.57 (1.06, 2.32)	0.0258
<=45 years +no MHT	29	8773	1.32 (0.84, 2.06)	0.2259
46-50 years + MHT	64	18,816	1.19 (0.83, 1.70)	0.35
46-50 years + no MHT	58	28,276	reference	
51-55 years +MHT	61	22,579	0.93 (0.65, 1.34)	0.7006
51-55 years +no MHT	59	36,454	0.76 (0.53, 1.09)	0.1345
>55 years+ MHT	21	6309	1.00 (0.60, 1.65)	0.9919
>55 years+ no MHT	16	7413	0.75 (0.43, 1.30)	0.3007

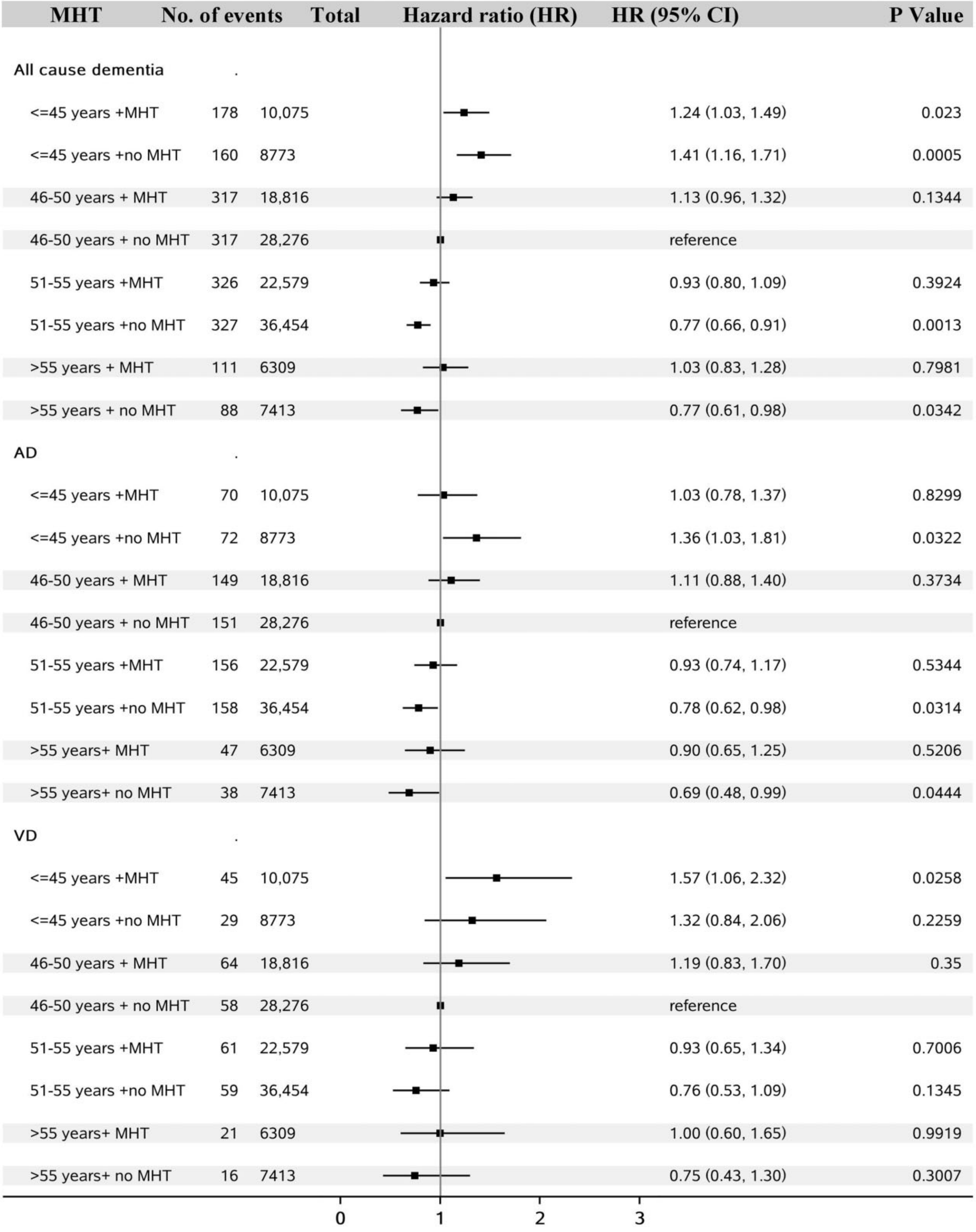

Supplementary Figure S1. Combined effect of age at menopause and MHT (menopausal hormone therapy) on risk of incident all-cause dementia, Alzheimer's dementia (AD), and vascular dementia (VD).
