# Supplementary material for: Age at menopause and all-cause and cause-specific dementia: a prospective analysis of the UK Biobank cohort
Source: Hum Reprod. 2023 Jun 21;38(9):1746–54. doi: 10.1093/humrep/dead130 (PMC10663050; doi:10.1093/humrep/dead130)
Supplement: dead130_Supplementary_Figure_S2 [file dead130_supplementary_figure_s2.pdf]

Human Reproduction, 2023, 1–1
https://doi.org/10.1093/humrep/dead130
Supplementary Data

Income levels	No. of events	Total	HR (95% CI)	P Value
**All cause dementia**	.			
<=45 years +Income < £ 18,000	175	5750	1.67 (1.36, 2.06)	<.0001
<=45 years +Income £ 18,000-30,999	80	4914	1.18 (0.91, 1.53)	0.2272
<=45 years +Income ≥ £ 31,000	83	8042	1.04 (0.8, 1.35)	0.7652
46-50 years +Income < £ 18,000	276	12,693	1.25 (1.04, 1.51)	0.0183
46-50 years +Income £ 18,000-30,999	161	12,609	0.93 (0.75, 1.14)	0.4663
46-50 years +Income ≥ £ 31,000	197	21,790	reference	
51-55 years + Income < £ 18,000	288	14,563	1.13 (0.94, 1.36)	0.1983
51-55 years + Income £ 18,000-30,999	177	15,975	0.79 (0.65, 0.97)	0.026
51-55 years + Income ≥ £ 31,000	188	28,495	0.70 (0.57, 0.86)	0.0006
>55 years+ Income < £ 18,000	92	3940	1.17 (0.91, 1.50)	0.2195
>55 years + Income £ 18,000-30,999	54	3858	0.85 (0.63, 1.15)	0.2794
>55 years+ Income ≥ £ 31,000	53	5924	0.65 (0.48, 0.88)	0.0059
**AD**	.			
<=45 years +Income < £ 18,000	68	5750	1.34 (0.97, 1.84)	0.074
<=45 years +Income £ 18,000-30,999	40	4914	1.20 (0.83, 1.75)	0.3275
<=45 years +Income ≥ £ 31,000	34	8042	0.93 (0.63, 1.37)	0.7063
46-50 years +Income < £ 18,000	127	12,693	1.18 (0.90, 1.55)	0.2286
46-50 years +Income £ 18,000-30,999	80	12,609	0.93 (0.69, 1.26)	0.6266
46-50 years +Income ≥ £ 31,000	93	21,790	reference	
51-55 years + Income < £ 18,000	133	14,563	1.06 (0.81, 1.39)	0.6796
51-55 years + Income £ 18,000-30,999	89	15,975	0.81 (0.60, 1.08)	0.1494
51-55 years + Income ≥ £ 31,000	92	28,495	0.73 (0.54, 0.97)	0.0309
>55 years+ Income < £ 18,000	39	3940	1.00 (0.68, 1.46)	0.9917
>55 years + Income £ 18,000-30,999	28	3858	0.89 (0.58, 1.36)	0.5829
>55 years+ Income ≥ £ 31,000	18	5924	0.45 (0.27, 0.76)	0.0026
**VD**	.			
<=45 years +Income < £ 18,000	40	5750	1.85 (1.17, 2.93)	0.0086
<=45 years +Income £ 18,000-30,999	19	4914	1.41 (0.81, 2.46)	0.2286
<=45 years +Income ≥ £ 31,000	15	8042	0.92 (0.50, 1.71)	0.7911
46-50 years +Income < £ 18,000	58	12,693	1.33 (0.87, 2.04)	0.1829
46-50 years +Income £ 18,000-30,999	28	12,609	0.85 (0.52, 1.40)	0.5275
46-50 years +Income ≥ £ 31,000	36	21,790	reference	
51-55 years + Income < £ 18,000	52	14,563	1.06 (0.69, 1.63)	0.7874
51-55 years + Income £ 18,000-30,999	35	15,975	0.82 (0.52, 1.31)	0.4103
51-55 years + Income ≥ £ 31,000	33	28,495	0.69 (0.43, 1.10)	0.121
>55 years+ Income < £ 18,000	17	3940	1.06 (0.59, 1.90)	0.8446
>55 years + Income £ 18,000-30,999	7	3858	0.55 (0.25, 1.24)	0.1511
>55 years+ Income ≥ £ 31,000	13	5924	0.85 (0.45, 1.61)	0.6225

Hazard ratio (HR)

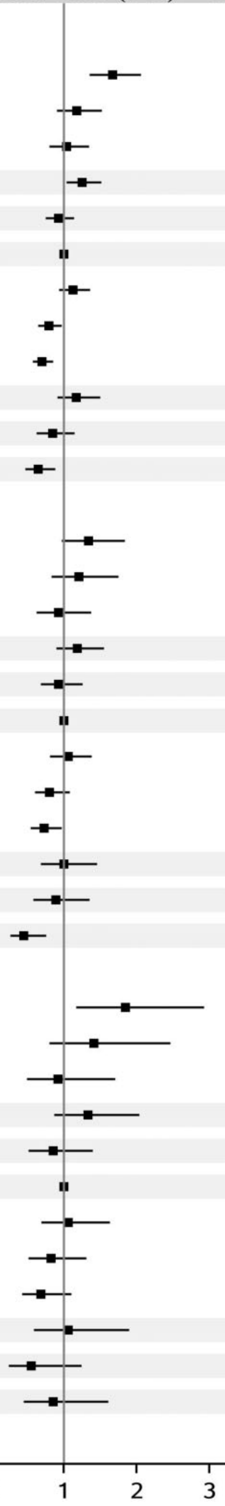

Supplementary Figure S2. Combined effect of age at menopause and income level on risk of incident all-cause dementia, Alzheimer's dementia (AD) and vascular dementia (VD).
