# Supplementary material for: Age at menopause and all-cause and cause-specific dementia: a prospective analysis of the UK Biobank cohort
Source: Hum Reprod. 2023 Jun 21;38(9):1746–54. doi: 10.1093/humrep/dead130 (PMC10663050; doi:10.1093/humrep/dead130)
Supplement: dead130_Supplementary_Figure_S3 [file dead130_supplementary_figure_s3.pdf]

OXFORD
human reproduction

Human Reproduction, 2023, 1–1
https://doi.org/10.1093/humrep/dead130
Supplementary Data

leisure activities	No. of events	Total	HR (95% CI)	P Value
**All cause dementia**				
<=45 years + no leisure activities	122	5601	1.68 (1.32, 2.14)	<.0001
<=45 years + one leisure activity	148	8012	1.47 (1.18, 1.85)	0.0008
<=45 years + two leisure activities	68	5093	1.11 (0.84, 1.48)	0.4628
46-50 years + no leisure activities	201	12,971	1.30 (1.05, 1.61)	0.0145
46-50 years + one leisure activity	275	19,823	1.18 (0.97, 1.44)	0.1026
46-50 years + two leisure activities	158	14,298	reference	
51-55 years + one leisure activity	285	24,402	1.15 (0.93, 1.42)	0.2064
51-55 years + no leisure activities	196	14,510	0.98 (0.80, 1.19)	0.8293
51-55 years + two leisure activities	172	20,121	0.76 (0.61, 0.94)	0.0121
>55 years + no leisure activities	55	3249	1.07 (0.78, 1.45)	0.6908
>55 years + one leisure activity	97	5660	1.10 (0.85, 1.41)	0.4867
>55 years + two leisure activities	47	4813	0.71 (0.51, 0.98)	0.038
**AD**				
<=45 years + no leisure activities	53	5601	1.44 (1.01, 2.05)	0.0432
<=45 years + one leisure activity	63	8012	1.23 (0.88, 1.71)	0.2261
<=45 years + two leisure activities	26	5093	0.82 (0.53, 1.28)	0.3803
46-50 years + no leisure activities	89	12,971	1.14 (0.84, 1.55)	0.3911
46-50 years + one leisure activity	128	19,823	1.05 (0.79, 1.38)	0.7574
46-50 years + two leisure activities	83	14,298	reference	
51-55 years + no leisure activities	90	24,402	1.00 (0.74, 1.36)	0.9852
51-55 years + one leisure activity	141	14,510	0.94 (0.71, 1.23)	0.6287
51-55 years + two leisure activities	83	20,121	0.70 (0.52, 0.95)	0.0239
>55 years + no leisure activities	19	3249	0.71 (0.43, 1.16)	0.171
>55 years + one leisure activity	43	5660	0.94 (0.65, 1.35)	0.7231
>55 years + two leisure activities	23	4813	0.64 (0.40, 1.03)	0.0674
**VD**				
<=45 years + no leisure activities	32	5601	1.98 (1.20, 3.27)	0.0078
<=45 years + one leisure activity	31	8012	1.44 (0.88, 2.37)	0.1481
<=45 years + two leisure activities	11	5093	0.86 (0.43, 1.70)	0.6601
46-50 years + no leisure activities	48	12,971	1.51 (0.96, 2.38)	0.0732
46-50 years + one leisure activity	42	19,823	0.88 (0.55, 1.40)	0.5829
46-50 years + two leisure activities	32	14,298	reference	
51-55 years + no leisure activities	34	24,402	0.97 (0.59, 1.57)	0.8868
51-55 years + one leisure activity	54	14,510	0.93 (0.60, 1.43)	0.7287
51-55 years + two leisure activities	32	20,121	0.71 (0.44, 1.16)	0.1742
>55 years + no leisure activities	9	3249	0.80 (0.38, 1.67)	0.5441
>55 years + one leisure activity	19	5660	1.01 (0.57, 1.78)	0.9796
>55 years + two leisure activities	9	4813	0.66 (0.32, 1.39)	0.278

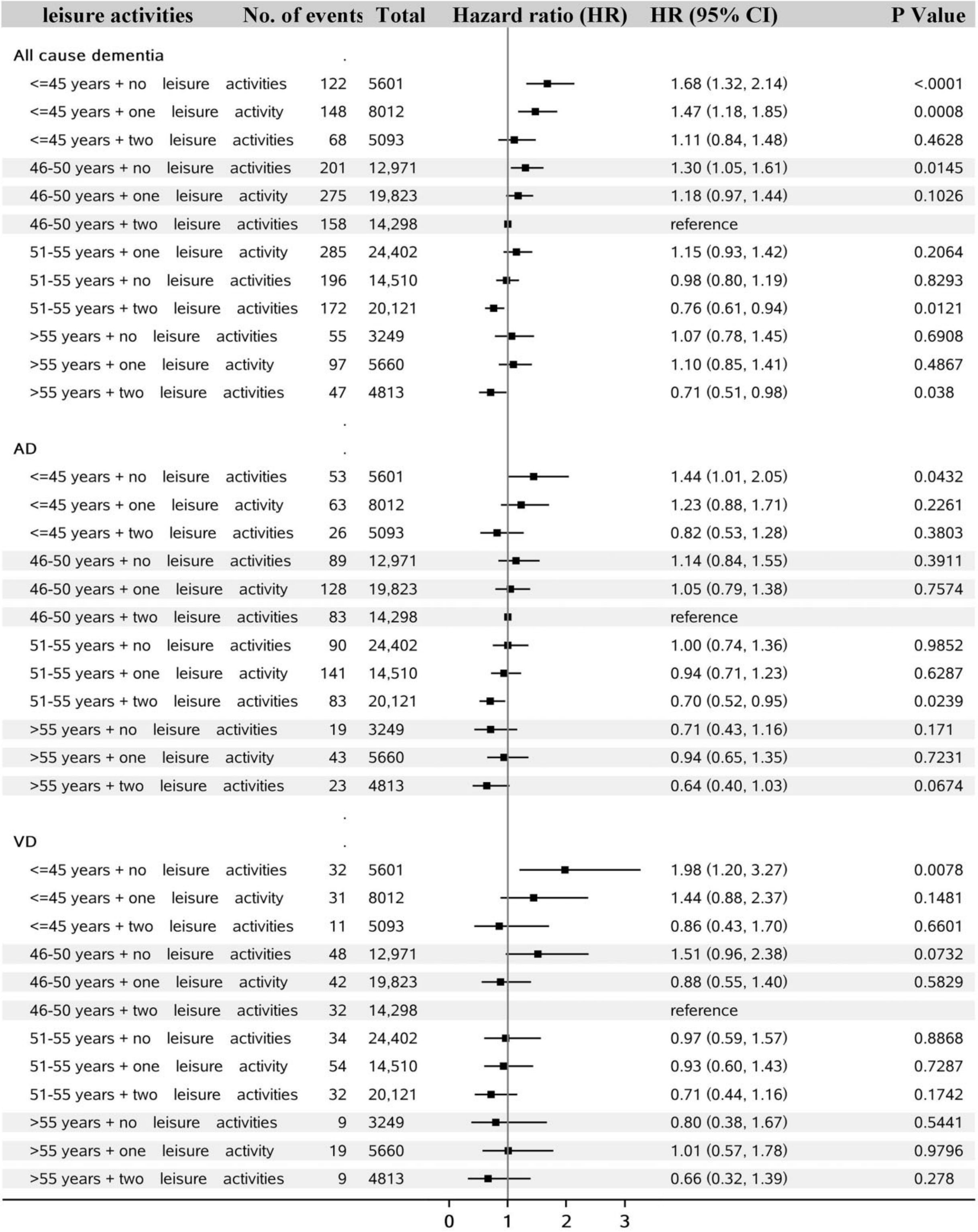

Supplementary Figure S3. Combined effect of age at menopause and leisure activities levels on risk of incident all-cause dementia, Alzheimer's dementia (AD) and vascular dementia (VD).
