# Supplementary material for: Age at menopause and all-cause and cause-specific dementia: a prospective analysis of the UK Biobank cohort
Source: Hum Reprod. 2023 Jun 21;38(9):1746–54. doi: 10.1093/humrep/dead130 (PMC10663050; doi:10.1093/humrep/dead130)
Supplement: dead130_Supplementary_Table_S1 [file dead130_supplementary_table_s1.pdf]

OXFORD
human reproduction

Human Reproduction, 2023, 1–1
https://doi.org/10.1093/humrep/dead130
Supplementary Data

Supplementary Table S1. Exposures, outcomes, and covariates' definitions and descriptions.

Variables	Categorizations	UK Biobank code	Descriptions
**Age at natural menopause**	≤40 years,<br>41–45 years,<br>46–50 years,<br>51–55 years,<br>≥55 years	3581, 2724	Touchscreen question<br>(i) 'How old were you when your periods stopped?'<br>(ii) 'Have you had your menopause (periods stopped)?'
**Age at surgical menopause**	≤40 years,<br>41–45 years,<br>46–50 years,<br>51–55 years,<br>≥55 years	3882	Touchscreen question 'How old were you when you had BOTH ovaries removed?'
**All cause dementia**	No,<br>Yes	ICD-10 codes: F00, F01, F02, F03, G30, G310, G311, G318;<br>ICD-9 codes: 290	ICD-10 codes:<br>F00 Dementia in Alzheimer's disease<br>F01 Vascular dementia<br>F02 Dementia in other diseases classified elsewhere<br>F03 Unspecified dementia<br>G30 Alzheimer's disease<br>G310 Circumscribed brain atrophy<br>G311 Senile degeneration of brain, not elsewhere classified<br>G318 Other specified degenerative disease of nervous system<br>ICD-9 code 290: Senile and presenile organic psychotic conditions
**Alzheimer**	No,<br>Yes	ICD-10 codes: F00, G30;<br>ICD-9 codes: 290.1	ICD-10 codes:<br>F00 Dementia in Alzheimer's disease<br>G30 Alzheimer's disease<br>ICD-9 code 290: Senile and presenile organic psychotic conditions
**Vascular dementia**	No,<br>Yes	ICD-10 codes: F01	ICD-10 codes:<br>F01 Vascular dementia
**Age (years)**	Age at baseline	21022	Date attended baseline assessment minus date of birth
**Ethnicity**	White, Asian or Asian British, Black or Black British, Other	21000	Touchscreen questionnaire: 'What is your ethnic group?'
**Education levels**	Years of education ≤10,<br>11–12,<br>>12	6138, 845	Touchscreen questionnaire:<br>(i) 'Which of the following qualifications do you have?'<br>(ii) 'At what age did you complete your continuous full-time education?'
**Income levels**	$Level_1$: Less than £18 000<br>$Level_2$: £18 000–30 999<br>$Level_3$: greater than £31 000	738	Touchscreen questionnaire: 'what is the average total income before tax received by your HOUSEHOLD?'
**BMI (kg/m^2)**	Underweight < 18.5,<br>Normal (18.5, 24.9),<br>Overweight (25.0, 29.9),<br>Obese ≥ 30.0	21001	Physical examination: BMI
**Smoking status**	Never,<br>Former,<br>Current	20116	Touchscreen questionnaire: 'How often do you smoke tobacco?'
**Alcohol intake**	Daily or almost daily,<br>3–4 times a week,<br>1–2 times a week,<br>Occasionally,<br>Never	1558	Touchscreen questionnaire: 'About how often do you drink alcohol?'
**Leisure/social activities**	Sports club or gym,<br>Pub or social club,<br>Religious group,<br>Adult education class,<br>Other group activity,<br>None of the above,	6160	Touchscreen question 'Which of the following do you attend once a week or more often? (You can select more than one)'

(continued)

Supplementary Table S1. Continued

Variables	Categorizations	UK Biobank code	Descriptions
**Physical activities**	Low,<br>Moderate,<br>High	22032	UK Biobank used International Physical Activity Questionnaire (IPAQ) to calculate metabolic equivalent (MET) score. For those who were missing with MET score, we used their number of days/week of moderate physical activity to categorize their physical activity level.
**Cardiovascular disease (CVD)**	No,<br>Yes	6150	Touchscreen questionnaire and verbal interview: 'Do you regularly take any of the following medications?'
**APOE e4 carrier status**	No APOE e4,<br>One APOE e4,<br>Two APOE e4	rs7412, rs429358	Number of APOE e4: none (e2/e2, e2/e3, or e3/e3 haplotypes), one (e3/e4 and occasionally e2/e4 haplotypes), and two (e4/e4 haplotypes)
**Menopausal hormone therapy (MHT)**	Yes,<br>No	2814	Touchscreen question 'Have you ever used hormone replacement therapy (HRT)?'
**Age at natural menopause**	≤40 years,<br>41–45 years,<br>46–50 years,<br>51–55 years,<br>≥55 years	3581, 2724	Touchscreen question<br>(i) 'How old were you when your periods stopped?'<br>(ii) 'Have you had your menopause (periods stopped)?'
**Age at surgical menopause**	≤40 years,<br>41–45 years,<br>46–50 years,<br>51–55 years,<br>≥55 years	3882	Touchscreen question 'How old were you when you had BOTH ovaries removed?'
**All cause dementia**	No,<br>Yes	ICD-10 codes: F00, F01, F02, F03, G30, G310, G311, G318;<br>ICD-9 codes: 290	ICD-10 codes:<br>F00 Dementia in Alzheimer's disease<br>F01 Vascular dementia<br>F02 Dementia in other diseases classified elsewhere<br>F03 Unspecified dementia<br>G30 Alzheimer's disease<br>G310 Circumscribed brain atrophy<br>G311 Senile degeneration of brain, not elsewhere classified<br>G318 Other specified degenerative disease of nervous system<br>ICD-9 code 290: Senile and presenile organic psychotic conditions
**Alzheimer**	No,<br>Yes	ICD-10 codes: F00, G30;<br>ICD-9 codes: 290.1	ICD-10 codes:<br>F00 Dementia in Alzheimer's disease<br>G30 Alzheimer's disease<br>ICD-9 code 290: Senile and presenile organic psychotic conditions
**Vascular dementia**	No,<br>Yes	ICD-10 codes: F01	ICD-10 codes:<br>F01 Vascular dementia
**Age (years)**	Age at baseline	21022	Date attended baseline assessment minus date of birth
**Ethnicity**	White, Asian or Asian British, Black or Black British, Other	21000	Touchscreen questionnaire: 'What is your ethnic group?'
**Education levels**	Years of education ≤10,<br>11–12,<br>>12	6138, 845	Touchscreen questionnaire:<br>(i) 'Which of the following qualifications do you have?'<br>(ii) 'At what age did you complete your continuous full-time education?'
**Income levels**	$Level_1$: Less than £18 000<br>$Level_2$: £18 000–30 999<br>$Level_3$: greater than £31 000	738	Touchscreen questionnaire: 'what is the average total income before tax received by your HOUSEHOLD?'
**BMI (kg/m^2)**	Underweight < 18.5,<br>Normal (18.5, 24.9),<br>Overweight (25.0, 29.9),<br>Obese ≥ 30.0	21001	Physical examination: BMI
**Smoking status**	Never,<br>Former,<br>Current	20116	Touchscreen questionnaire: 'How often do you smoke tobacco?'
**Alcohol intake**	Daily or almost daily,<br>3–4 times a week,<br>1–2 times a week,<br>Occasionally,<br>Never	1558	Touchscreen questionnaire: 'About how often do you drink alcohol?'
**Leisure/social activities**	Sports club or gym,<br>Pub or social club,<br>Religious group,<br>Adult education class,<br>Other group activity,<br>None of the above,	6160	Touchscreen question 'Which of the following do you attend once a week or more often? (You can select more than one)'

(continued)

Supplementary Table S1. Continued

Variables	Categorizations	UK Biobank code	Descriptions
**Physical activities**	Low, Moderate, High	22032	UK Biobank used International Physical Activity Questionnaire (IPAQ) to calculate metabolic equivalent (MET) score. For those who were missing with MET score, we used their number of days/ week of moderate physical activity to categorize their physical activity level.
**Cardiovascular disease (CVD)**	No, Yes	6150	Touchscreen questionnaire and verbal interview: 'Do you regularly take any of the following medications?'
**APOE e4 carrier status**	No APOE e4, One APOE e4, Two APOE e4	rs7412, rs429358	Number of APOE e4: none (e2/e2, e2/e3, or e3/e3 haplotypes), one (e3/e4 and occasionally e2/e4 haplotypes), and two (e4/e4 haplotypes)
**Menopausal hormone therapy (MHT)**	Yes, No	2814	Touchscreen question 'Have you ever used hormone replacement therapy (HRT)?'
