# Supplementary material for: Age at menopause and all-cause and cause-specific dementia: a prospective analysis of the UK Biobank cohort
Source: Hum Reprod. 2023 Jun 21;38(9):1746–54. doi: 10.1093/humrep/dead130 (PMC10663050; doi:10.1093/humrep/dead130)
Supplement: dead130_Supplementary_Table_S2 [file dead130_supplementary_table_s2.pdf]

Human Reproduction, 2023, 1–1
https://doi.org/10.1093/humrep/dead130
Supplementary Data

Supplementary Table S2. Characteristics by age at surgical menopause and incident all-cause dementia events.

Characteristics	Age at surgical menopause					Incident dementia	
	≤40 years (n = 2862)	41–45 years (n = 4945)	46–50 years (n = 6238)	51–55 years (n = 3754)	≥55 years (n = 3728)	No (n = 21 167)	Yes (n = 360)
Age at baseline	56.3 ± 7.9	57.1 ± 6.9	59.3 ± 6.0	60.7 ± 5.3	63.4 ± 4.2	59.3 ± 6.6	64.1 ± 5.0
Ethnicity							
White	2715 (94.9)	4679 (94.6)	5964 (95.6)	3615 (96.3)	3607 (96.8)	20 241 (95.6)	339 (94.2)
Nonwhite	147 (5.1)	266 (5.4)	274 (4.4)	139 (3.7)	121 (3.2)	926 (4.4)	21 (5.8)
Education level							
≤10	1783 (62.3)	2938 (59.4)	3584 (57.5)	1968 (52.4)	2145 (57.5)	12 176 (57.5)	242 (67.2)
11–12	333 (11.6)	552 (11.2)	675 (10.8)	455 (12.1)	360 (9.7)	2338 (11.2)	37 (10.3)
>12	746 (26.1)	1455 (29.4)	1979 (31.7)	1331 (35.5)	1223 (32.8)	6653 (31.3)	81 (22.5)
Income (£)							
<18 000	879 (30.7)	1426 (28.8)	1809 (29.0)	1082 (28.8)	1251 (33.6)	6299 (29.8)	148 (41.1)
18 000–30 999	708 (24.7)	1255 (25.4)	1764 (28.3)	1051 (28.0)	1089 (29.2)	5767 (27.2)	100 (27.8)
≥31 000	1275 (44.6)	2264 (45.8)	2665 (42.7)	1621 (43.2)	1388 (37.2)	9101 (43.0)	112 (31.1)
BMI							
<18.5 kg/m^2	18 (0.6)	15 (0.3)	37 (0.6)	13 (0.4)	24 (0.6)	103 (0.5)	4 (1.1)
18.5–24.9 kg/m^2	785 (27.4)	1440 (29.2)	2006 (32.2)	1282 (34.2)	1204 (32.3)	6615 (31.2)	102 (28.3)
25.0–29.9 kg/m^2	1075 (37.6)	1945 (39.3)	2396 (38.4)	1440 (38.4)	1437 (38.6)	8140 (38.5)	153 (42.5)
≥30kg/m^2	984 (34.4)	1545 (31.2)	1799 (28.8)	1019 (27.0)	1063 (28.5)	6309 (29.8)	101 (28.1)
Cigarette smoking							
Never smoker	1481 (51.8)	2794 (56.5)	3634 (58.3)	2302 (61.3)	2148 (57.6)	12 166 (57.5)	193 (53.6)
Former smoker	928 (32.4)	1612 (32.6)	2132 (34.2)	1233 (32.8)	1352 (36.3)	7132 (33.7)	125 (34.7)
Current smoker	453 (15.8)	539 (10.9)	472 (7.5)	219 (5.9)	228 (6.1)	1869 (8.8)	42 (11.7)
Alcohol drinking							
Never drinker	248 (8.7)	328 (6.6)	368 (5.9)	218 (5.8)	264 (7.1)	1391 (6.6)	35 (9.7)
Former drinker	196 (6.7)	256 (5.2)	271 (4.3)	118 (3.1)	156 (4.2)	970 (4.6)	27 (7.5)
Current drinker	2418 (84.6)	4361 (88.2)	5599 (89.8)	3418 (91.1)	3308 (88.7)	18 806 (88.8)	298 (82.8)
No. of leisure activities							
0	1028 (35.9)	1583 (32.0)	1808 (29.0)	1057 (28.2)	991 (26.6)	6339 (30.0)	128 (35.6)
1	1144 (40.0)	2055 (41.6)	2627 (42.1)	1516 (40.4)	1547 (41.5)	8732 (41.3)	157 (43.6)
≥2	690 (24.1)	1307 (26.4)	1803 (28.9)	1181 (31.5)	1190 (31.9)	6096 (28.7)	75 (20.8)
CVD							
No	2555 (89.3)	4575 (92.5)	5802 (93.0)	3499 (93.2)	3380 (90.7)	19 533 (92.3)	278 (77.2)
Yes	307 (10.7)	370 (7.5)	436 (7.0)	255 (6.8)	348 (9.3)	1634 (7.7)	82 (22.8)
APOE e4							
No APOE e4	2175 (76.0)	3717 (75.2)	4697 (75.3)	2775 (73.9)	2892 (77.6)	16 081 (76.0)	175 (48.6)
One APOE e4	615 (21.5)	1120 (22.7)	1412 (22.6)	899 (24.0)	772 (20.7)	4667 (22.1)	151 (42.0)
Two APOE e4	72 (2.5)	108 (2.1)	129 (2.1)	80 (2.1)	64 (1.7)	419 (1.9)	34 (9.4)
Ever-used MHT at baseline							
No	391 (13.7)	575 (11.6)	939 (15.1)	1016 (27.1)	1367 (36.7)	4192 (19.8)	96 (26.7)
Yes	2471 (86.3)	4370 (88.4)	5299 (84.9)	2738 (72.9)	2361 (63.3)	16 975 (80.2)	264 (73.3)

CVD, cardiovascular disease; APOE, apolipoprotein E; MHT, menopausal hormone therapy.
