# Supplementary material for: Age at menopause and all-cause and cause-specific dementia: a prospective analysis of the UK Biobank cohort
Source: Hum Reprod. 2023 Jun 21;38(9):1746–54. doi: 10.1093/humrep/dead130 (PMC10663050; doi:10.1093/humrep/dead130)
Supplement: dead130_Supplementary_Table_S3 [file dead130_supplementary_table_s3.pdf]

OXFORD
human reproduction
Human Reproduction, 2023, 1–1
https://doi.org/10.1093/humrep/dead130
Supplementary Data

Supplementary Table S3. Baseline characteristics between women with and without (were excluded) information on age at menopause (%).

Characteristics	With information on age at menopause (n = 138 533)	Without information on age at menopause (n = 19 384)
Age at baseline (mean ± SD)	59.2 ± 6.1	61.7 ± 5.5
Ethnicity		
White	132 905 (95.9)	18 512 (95.5)
Nonwhite	5648 (4.1)	872 (4.5)
Education level		
≤10	69 130 (49.9)	11 274 (58.2)
11–12	16 604 (12.0)	2090 (10.8)
>12	52 819 (38.1)	6020 (31.1)
Income (£)		
<18 000	36 946 (26.7)	6524 (33.6)
18 000–30 999	37 356 (27.0)	5349 (27.6)
≥31 000	64 251 (46.3)	7511 (38.8)
BMI		
<18.5 kg/m^2	1092 (0.8)	132 (0.7)
18.5–24.9 kg/m^2	54 955 (39.7)	6739 (34.8)
25.0–29.9 kg/m^2	52 060 (37.6)	7661 (39.5)
≥30kg/m^2	30 446 (21.9)	4852 (25.0)
Cigarette smoking		
Never smoker	81 272 (58.7)	11 066 (57.1)
Former smoker	46 170 (33.3)	6856 (35.4)
Current smoker	11 111 (8.0)	1462 (7.5)
Alcohol drinking		
Never drinker	7559 (5.5)	1318 (6.8)
Former drinker	4840 (3.5)	839 (4.3)
Current drinker	126 154 (91.0)	17 227 (88.9)
No. of leisure activities		
0	36 331 (26.2)	5470 (28.2)
1	57 897 (41.8)	8162 (42.1)
≥2	44 325 (32.0)	5752 (29.7)
CVD		
No	131 086 (94.6)	17 687 (91.2)
Yes	7467 (5.4)	1697 (8.8)
APOE e4		
No APOE e4,	105 127 (75.9)	14 609 (75.4)
One APOE e4,	30 661 (22.1)	4379 (22.6)
Two APOE e4	2765 (2.0)	396 (2.0)
MHT		
No	80 844 (58.4)	6725 (34.7)
Yes	57 709 (41.6)	12 659 (65.3)
