# Supplementary material for: Age at menopause and all-cause and cause-specific dementia: a prospective analysis of the UK Biobank cohort
Source: Hum Reprod. 2023 Jun 21;38(9):1746–54. doi: 10.1093/humrep/dead130 (PMC10663050; doi:10.1093/humrep/dead130)
Supplement: dead130_Supplementary_Table_S4 [file dead130_supplementary_table_s4.pdf]

OXFORD
human reproduction

Human Reproduction, 2023, 1–1
https://doi.org/10.1093/humrep/dead130
Supplementary Data

Supplementary Table S4. Comparison dementia status and age at menopause between women who were excluded for missing covariates and who were included in the analytical sample (%).

	Without missing covariates (n = 138 553)	Missing covariates (n = 7525)
Age at natural menopause (year)	50.5 ± 4.5	50.0 ± 4.7
≤40 years	2427 (1.8)	255 (3.4)
41–45 years	16 279 (11.8)	1123 (14.9)
46–50 years	47 092 (34.0)	2671 (35.5)
51–55 years	59 033 (42.6)	2679 (35.6)
≥55 years	13 722 (9.8)	797 (10.6)
All cause dementia		
No	136 729 (98.7)	7343 (97.8)
Yes	1824 (1.3)	182 (2.2)
