# Supplementary material for: Age at menopause and all-cause and cause-specific dementia: a prospective analysis of the UK Biobank cohort
Source: Hum Reprod. 2023 Jun 21;38(9):1746–54. doi: 10.1093/humrep/dead130 (PMC10663050; doi:10.1093/humrep/dead130)
Supplement: dead130_Supplementary_Table_S5 [file dead130_supplementary_table_s5.pdf]

OXFORD

human reproduction

Human Reproduction, 2023, 1–1

https://doi.org/10.1093/humrep/dead130

Supplementary Data

Supplementary Table S5. Associations between age at natural menopause (including premenopausal) and all-cause dementia, Alzheimer's disease (AD) and vascular dementia (VD).

Dementia	Years	Women	Dementia	Incidence rate (per 10 000 person-years)	Model 1 HR (95% CI)	Model 2 HR (95% CI)	Model 3 HR (95% CI)	Model 4 HR (95% CI)
	Premenopausal	72 234	144	1.6	1.14 (0.91, 1.43)	1.14 (0.92, 1.43)	1.15 (0.92, 1.44)	1.16 (0.93, 1.44)
	≤40	2390	49	16.8	1.59 (1.19, 2.14)	1.50 (1.12, 2.01)	1.36 (1.01, 1.83)	1.35 (1.00, 1.81)
	41–45	15 835	289	15.0	1.25 (1.08, 1.43)	1.23 (1.07, 1.41)	1.19 (1.04, 1.37)	1.18 (1.03, 1.36)
All-cause dementia	46–50	44 755	633	11.5	1	1	1	1
	51–55	54 587	653	9.7	0.80 (0.72, 0.90)	0.82 (0.74, 0.92)	0.83 (0.75, 0.93)	0.83 (0.75, 0.93)
	>55	12 684	199	12.9	0.81 (0.69, 0.96)	0.84 (0.71, 0.98)	0.84 (0.72, 0.99)	0.84 (0.71, 0.99)
	Premenopausal	72 234	46	0.5	0.95 (0.66, 1.36)	0.95 (0.66, 1.36)	0.96 (0.67, 1.37)	0.96 (0.67, 1.37)
	≤40	2390	24	8.4	1.71 (1.13, 2.60)	1.63 (1.07, 2.47)	1.49 (0.98, 2.27)	1.48 (0.98, 2.25)
	41–45	15 835	118	6.1	1.08 (0.87, 1.34)	1.06 (0.86, 1.32)	1.04 (0.84, 1.29)	1.04 (0.84, 1.29)
Alzheimer's disease (AD)	46–50	44 755	299	5.5	1	1	1	1
	51–55	54 587	314	4.7	0.82 (0.70, 0.96)	0.84 (0.71, 0.98)	0.85 (0.72, 0.99)	0.85 (0.72, 0.99)
	>55	12 684	85	5.5	0.73 (0.58, 0.94)	0.75 (0.59, 0.96)	0.75 (0.59, 0.96)	0.75 (0.59, 0.96)
	Premenopausal	72 234	22	0.2	1.35 (0.80, 2.28)	1.34 (0.80, 2.26)	1.34 (0.80, 2.24)	1.34 (0.80, 2.25)
	≤40	2390	12	4.2	1.99 (1.10, 3.60)	1.84 (1.01, 3.33)	1.61 (0.89, 2.92)	1.59 (0.87, 2.88)
	41–45	15 835	62	3.2	1.34 (0.98, 1.82)	1.31 (0.96, 1.78)	1.26 (0.92, 1.71)	1.25 (0.91, 1.70)
Vascular dementia (VD)	46–50	44 755	122	2.2	1	1	1	1
	51–55	54 587	120	1.8	0.77 (0.59, 0.99)	0.79 (0.61, 1.02)	0.80 (0.62, 1.03)	0.80 (0.62, 1.03)
	>55	12 684	37	2.4	0.74 (0.51, 1.08)	0.77 (0.53, 1.11)	0.78 (0.54, 1.13)	0.78 (0.54, 1.12)

Model 1: adjusted for age at baseline, race, BMI, education level, income level; Model 2: Model 1 plus leisure activities, cigarette smoking, alcohol drinking; Model 3: Model 2 plus CVD (Cardiovascular disease) and APOE (apolipoprotein E); Model 4: Model 3 plus ever-used menopausal hormone therapy (MHT) at baseline. HR, hazard ratio; CI, confidence interval.
