# Supplementary material for: Age at menopause and all-cause and cause-specific dementia: a prospective analysis of the UK Biobank cohort
Source: Hum Reprod. 2023 Jun 21;38(9):1746–54. doi: 10.1093/humrep/dead130 (PMC10663050; doi:10.1093/humrep/dead130)
Supplement: dead130_Supplementary_Table_S6 [file dead130_supplementary_table_s6.pdf]

OXFORD

human reproduction

Human Reproduction, 2023, 1–1
https://doi.org/10.1093/humrep/dead130
Supplementary Data

Supplementary Table S6. The associations between age at natural menopause and dementia with cardiovascular diseases (CVD) being adjusted or not.

Age at natural menopause	All cause dementia		Alzheimer's Disease		Vascular dementia (VD)	
	CVD not adjusted	CVD adjusted	CVD not adjusted	CVD adjusted	CVD not adjusted	CVD adjusted
≤40	1.44 (1.08, 1.94)	1.36 (1.01, 1.83)	1.56 (1.03, 2.36)	1.48 (0.98, 2.25)	1.74 (0.96, 3.16)	1.59 (0.88, 2.88)
41–45	1.22 (1.06, 1.40)	1.19 (1.03, 1.37)	1.05 (0.85, 1.31)	1.04 (0.84, 1.29)	1.29 (0.95, 1.76)	1.25 (0.92, 1.70)
46–50	1	1	1	1	1	1
51–55	0.82 (0.73, 0.91)	0.83 (0.74, 0.92)	0.83 (0.71, 0.98)	0.84 (0.72, 0.98)	0.79 (0.61, 1.01)	0.80 (0.62, 1.03)
>55	0.83 (0.71, 0.97)	0.83 (0.71, 0.98)	0.74 (0.58, 0.95)	0.74 (0.58, 0.95)	0.77 (0.53, 1.11)	0.78 (0.54, 1.12)

All models were adjusted age at baseline, race, BMI education level, income level, leisure activities, cigarette smoking, alcohol drinking, and APOE (apolipoprotein E), ever-used menopausal hormone therapy (MHT) at baseline.
