# Supplementary material for: Age at menopause and all-cause and cause-specific dementia: a prospective analysis of the UK Biobank cohort
Source: Hum Reprod. 2023 Jun 21;38(9):1746–54. doi: 10.1093/humrep/dead130 (PMC10663050; doi:10.1093/humrep/dead130)
Supplement: dead130_Supplementary_Table_S7 [file dead130_supplementary_table_s7.pdf]

OXFORD

human reproduction

Human Reproduction, 2023, 1–1
https://doi.org/10.1093/humrep/dead130
Supplementary Data

Supplementary Table S7. Associations between age at menopause and all-cause dementia, Alzheimer's disease (AD) and vascular dementia (VD): only included dementia events which occurred at least 5 years after menopause.

Dementia	Age at menopause (years)	Women (n)	Dementia events (n)	Model 1 HR (95% CI)	Model 2 HR (95% CI)	Model 3 HR (95% CI)	Model 4 HR (95% CI)
All-cause dementia	≤40	2427	49	1.60 (1.20, 2.15)	1.51 (1.13, 2.03)	1.37 (1.02, 1.84)	1.36 (1.01, 1.83)
	41–45	16 279	289	1.25 (1.09, 1.44)	1.23 (1.07, 1.42)	1.20 (1.04, 1.38)	1.19 (1.03, 1.37)
	46–50	47 087	629	1	1	1	1
	51–55	59 027	647	0.79 (0.71, 0.89)	0.81 (0.73, 0.91)	0.82 (0.74, 0.92)	0.82 (0.74, 0.92)
	>55	13 722	199	0.81 (0.69, 0.95)	0.83 (0.71, 0.98)	0.84 (0.71, 0.98)	0.83 (0.71, 0.98)
Alzheimer's disease (AD)	≤40	2427	24	1.71 (1.12, 2.59)	1.63 (1.07, 2.47)	1.49 (0.98, 2.26)	1.48 (0.98, 2.25)
	41–45	16 279	118	1.08 (0.87, 1.34)	1.06 (0.86, 1.32)	1.04 (0.84, 1.29)	1.04 (0.84, 1.29)
	46–50	47 087	299	1	1	1	1
	51–55	59 027	312	0.81 (0.69, 0.95)	0.83 (0.7, 0.97)	0.84 (0.71, 0.98)	0.84 (0.71, 0.98)
	>55	13 722	85	0.73 (0.57, 0.93)	0.74 (0.58, 0.95)	0.74 (0.58, 0.95)	0.74 (0.58, 0.95)
Vascular dementia (VD)	≤40	2427	12	1.98 (1.09, 3.58)	1.83 (1.01, 3.31)	1.61 (0.89, 2.92)	1.59 (0.88, 2.88)
	41–45	16 279	62	1.34 (0.99, 1.83)	1.31 (0.96, 1.78)	1.26 (0.93, 1.72)	1.25 (0.92, 1.71)
	46–50	47 087	121	1	1	1	1
	51–55	59 027	120	0.76 (0.59, 0.98)	0.79 (0.61, 1.01)	0.80 (0.62, 1.03)	0.80 (0.62, 1.03)
	>55	13 722	37	0.75 (0.52, 1.08)	0.77 (0.53, 1.12)	0.78 (0.54, 1.13)	0.78 (0.54, 1.12)

Model 1: adjusted for age at baseline, race, BMI, education level, income level; Model 2: Model 1 plus leisure activities, cigarette smoking, alcohol drinking; Model 3: Model 2 plus CVD (Cardiovascular disease) and APOE (apolipoprotein E); Model 4: Model 3 plus ever-used menopausal hormone therapy (MHT) at baseline. HR, hazard ratio; CI, confidence interval.
