# Supplementary material for: Age at menopause and all-cause and cause-specific dementia: a prospective analysis of the UK Biobank cohort
Source: Hum Reprod. 2023 Jun 21;38(9):1746–54. doi: 10.1093/humrep/dead130 (PMC10663050; doi:10.1093/humrep/dead130)
Supplement: dead130_Supplementary_Table_S8 [file dead130_supplementary_table_s8.pdf]

OXFORD
human reproduction

Human Reproduction, 2023, 1–1
https://doi.org/10.1093/humrep/dead130
Supplementary Data

Supplementary Table S8. Associations between age at natural menopause and all-cause dementia, Alzheimer's disease (AD) and vascular dementia (VD) in women without using MHT.

Dementia	Years	Women	Dementia	Incidence rate (per 10 000 person-years)	Model 1 HR (95% CI)	Model 2 HR (95% CI)	Model 3 HR (95% CI)
	≤40	802	15	14.9	1.21 (0.71, 2.06)	1.15 (0.67, 1.97)	0.98 (0.57, 1.68)
	41–45	7899	145	15.1	1.44 (1.18, 1.76)	1.42 (1.16, 1.73)	1.38 (1.13, 1.69)
All-cause dementia	46–50	28 276	317	9.0	1	1	1
	51–55	36 454	327	7.2	0.78 (0.67, 0.92)	0.80 (0.68, 0.94)	0.81 (0.69, 0.95)
	>55	7413	88	9.6	0.75 (0.59, 0.95)	0.77 (0.60, 0.98)	0.77 (0.61, 0.98)
	≤40	802	9	9.6	1.62 (0.82, 3.18)	1.55 (0.79, 3.04)	1.37 (0.69, 2.69)
	41–45	7899	63	6.6	1.30 (0.97, 1.75)	1.29 (0.96, 1.73)	1.26 (0.94, 1.70)
Alzheimer's disease (AD)	46–50	28 276	151	4.3	1	1	1
	51–55	36 454	158	3.5	0.80 (0.64, 1.00)	0.81 (0.65, 1.01)	0.82 (0.65, 1.03)
	>55	7413	38	4.1	0.68 (0.47, 0.97)	0.69 (0.48, 0.99)	0.69 (0.48, 0.99)
	≤40	802	5	5.3	2.17 (0.87, 5.41)	2.04 (0.82, 5.11)	1.64 (0.65, 4.12)
	41–45	7899	24	2.5	1.25 (0.78, 2.01)	1.22 (0.76, 1.97)	1.18 (0.73, 1.90)
Vascular dementia (VD)	46–50	28 276	58	1.7	1	1	1
	51–55	36 454	59	1.3	0.77 (0.53, 1.10)	0.79 (0.55, 1.13)	0.79 (0.55, 1.14)
	>55	7413	16	1.8	0.70 (0.4, 1.22)	0.72 (0.41, 1.26)	0.73 (0.42, 1.28)

Model 1: adjusted for age at baseline, race, BMI, education level, income level; Model 2: Model 1 plus leisure activities, cigarette smoking, alcohol drinking; Model 3: Model 2 plus CVD (cardiovascular disease) and APOE (apolipoprotein E). HR, hazard ratio; CI, confidence interval.
